# Advances in flexible graphene field-effect transistors for biomolecule sensing

**DOI:** 10.3389/fbioe.2023.1218024

**Published:** 2023-07-07

**Authors:** Bo Hu, Hao Sun, Jinpeng Tian, Jin Mo, Wantao Xie, Qiu Ming Song, Wenwei Zhang, Hui Dong

**Affiliations:** ^1^ Sino-German College of Intelligent Manufacturing, Shenzhen Technology University, Shenzhen, China; ^2^ School of Mechanical Engineering and Automation, Fuzhou University, Fuzhou, China

**Keywords:** flexible, graphene field-effect transistor, biomolecule, biosensor, biomarker

## Abstract

With the increasing demand for biomarker detection in wearable electronic devices, flexible biosensors have garnered significant attention. Additionally, graphene field-effect transistors (GFETs) have emerged as key components for constructing biosensors, owing to their high sensitivity, multifunctionality, rapid response, and low cost. Leveraging the advantages of flexible substrates, such as biocompatibility, adaptability to complex environments, and fabrication flexibility, flexible GFET sensors exhibit promising prospects in detecting various biomarkers. This review provides a concise summary of design strategies for flexible GFET biosensors, including non-encapsulated gate without dielectric layer coverage and external gate designs. Furthermore, notable advancements in sensing applications of biomolecules, such as proteins, glucose, and ions, are highlighted. Finally, we discuss the future challenges and prospects in this field, aiming to inspire researchers to address these issues in their further investigations.

## 1 Introduction

Graphene-base biosensors, including graphene field-effect transistors (GFETs), enable continuous label-free detect of biomarkers with exceptional sensitivity and selectivity. (Cai B J et al., 2014; Li Y J et al., 2017; Hao Z et al., 2018). GFETs also offer fast response times, making them well-suited for rapid detection of biomarkers. These features make GFET biosensors an ideal candidate for *in-vitro* detection of biomarkers.

Most of the GFET sensors uses a three-electrode structure, namely, drain, gate, and source electrodes. The drain and source electrodes are connected by graphene, which serves as the sensing element. Graphene holds a theoretical carrier mobility greater than 200,000 cm^2^ V^−1^ s^−1^ (Du X et al., 2008), which translates into high electrical conductivity in GFETs and enhances their sensitivity as sensors (Adzhri R et al., 2016). Furthermore, the high mechanical flexibility of graphene enables FETs to employed in wearable applications, expanding their potential utility. Graphene also functions as the biomolecule functioning sites. When biomolecules bind to the graphene surface, they modify the local charge density and electrical properties of the graphene, resulting in changes in the conductance of the GFET. These changes can be measured as a shift in the GFET’s transfer characteristic, enabling the detection and quantification of biomolecules (Tsang et al., 2019). By functionalizing GFETs with various types of biomolecules such as DNA, proteins, and aptamers, a wide range of analytes including ions (Sriram B et al., 2019; Tseliou F et al., 2019; Feng J et al., 2020), proteins (Manavalan S et al., 2019; Ku M et al., 2020; Seo G et al., 2020), bacteria (Jampasa et al., 2019; Chen T W et al., 2020) and other kinds of biomarkers (Zhan B et al., 2014; Bai Y et al., 2020) can be detected.

Flexible graphene field-effect transistor (F-GFET) biosensors are a new type of GFET sensor emerged recently (Meng S et al., 2021). Comparing with conventional GFET, which are typically fabricated on rigid and planar substrates like silicon (Jahromi A K et al., 2022). F-GFETs, on the other hand, are fabricated on flexible or stretchable substrates such as polyimide (Huang C et al., 2020), paralyene and other polymers (Kim S et al., 2022). Although rigid substrate provides a stable and flat surface for graphene deposition and device fabrication, which allows for precise control of device parameters and reproducibility, they lack flexibility, making them unsuitable for applications that require the use of conformal surfaces. Flexible substrates, however, provide the necessary mechanical flexibility. F-GFET offer several advantages over conventional GFET, such as conformal contact with biological tissues and improved device biocompatibility, which are critical for implantable and wearable devices. Additionally, F-GFETs enable real-time monitoring of physiological signals like muscle activity and heart rate, as well as environmental monitoring for gas sensing and humidity detection.

Currently, there is a paucity of literature reviews on flexible graphene biosensors, prompting us to author this comprehensive review article. This work summarizes the latest advancements in flexible graphene field-effect transistors (GFETs) for biomolecular sensing and identifies their limitations. It also presents future research prospects and solutions to address the current challenges in the field.

## 2 F-GFET biosensor design

Existing F-GFET device design mostly uses a liquid-gate configuration and can be further categorized into two sub-groups according to the gate electrode positions: the non-external gate (Hao Z et al., 2022) and the external gate (An J H et al., 2013).

**FIGURE 1:**
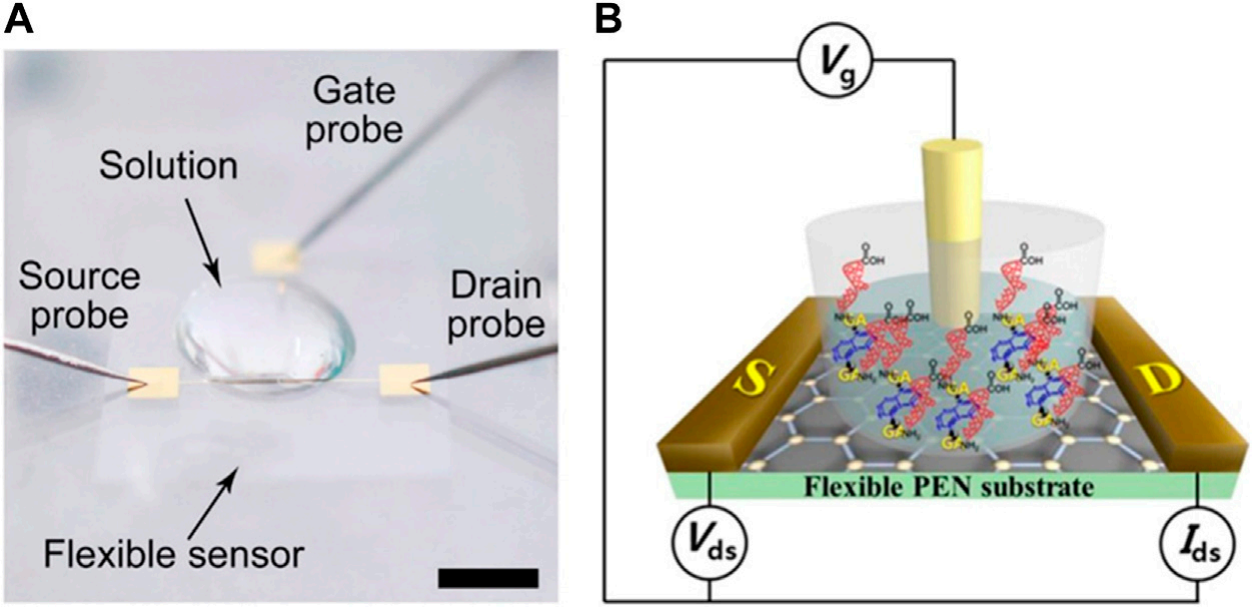
Flexible liquid-gated GFET **(A)** Non-encapsulated gate GFET without dielectric layer coverage. Adapted with permission from (Hao et al., 2022). Copyright© 2022, ACS. **(B)** External gate GFET. Adapted with permission from (An et al., 2013). Copyright© 2013, ACS.

In a typical flexible liquid gate GFET, graphene is used as the channel material for the source and drain. Chemical vapor deposition (CVD) is mainly used for synthesizing high-quality graphene, which is transferred to the substrates using either polymer-assisted transfer (Bahri M et al., 2021) or wet transfer methods (Hao Z et al., 2022). The electrical properties and biomarker responses of the F-GFET biosensor are investigated by introducing biological molecules of interest into the solution. The conductivity of the graphene channel in GFET can be modulated by changing the gate voltage (V_g_) at a constant drain-source voltage (V_ds_). Two main designs of gate electrode are commonly used: non-external and external. In the non-external design, the gate electrode is deposited onto the flexible substrate by metal deposition, the graphene channel region is directly exposed without any dielectric layer covering, making the graphene susceptible to environmental contamination, resulting in a degradation of GFET performance (Figure 1A). While in the external design, liquid electrodes such as Ag/AgCl or Pt electrodes are used for electrochemical sensing (Figure 1B).

## 3 Applications of F-GFET biosensors

Given graphene’s capability to undergo functionalization with different types of biomolecules, F-GFET holds significant promise for detecting an extensive array of analytes, encompassing DNA, proteins, small molecules, and various other substances. The following section categorizes the application of F-GFET by its analytes.

### 3.1 F-GFET for protein detection

**FIGURE 2:**
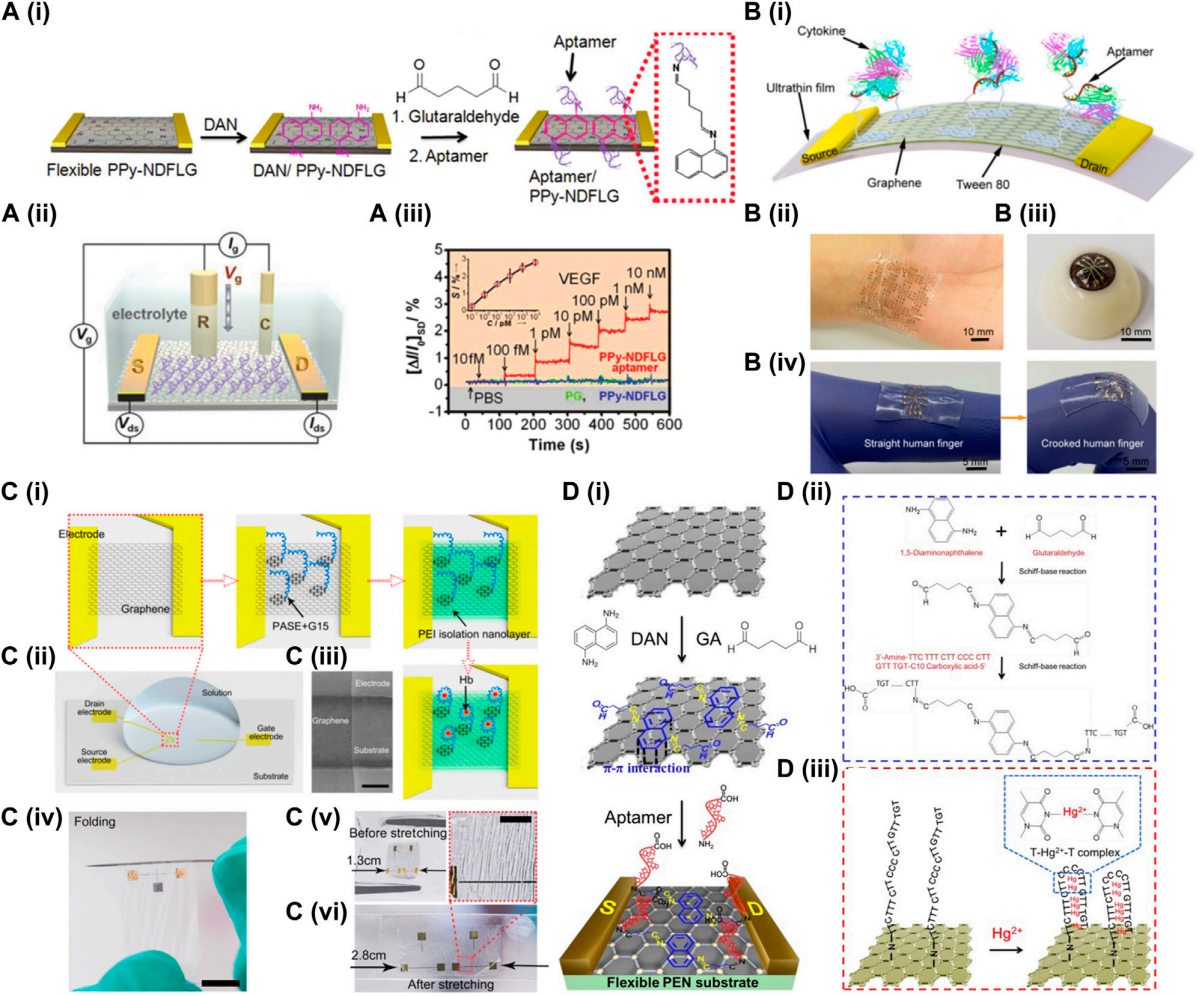
**(A)** (i) Schematic illustration of reaction steps for the fabrication of aptasensor platforms based on PPy-NDFLG conjugated with anti-VEGF RNA aptamer. (ii) Schematic diagram of a liquid-ion gated FET using aptamer-conjugated PPy-NDFLG (Ag/AgCl reference electrode, R; platinum counter electrode, C; source and drain electrodes, S and D). (iii) Real-time responses and a calibration curve (*S* in the inset indicates Δ*I*/*I*_0_) of aptasensor with various vascular endothelial growth factor concentrations. Adapted with permission from (Kwon et al., 2012). Copyright© 2012, ACS. **(B)** (i) Schematic of the GFET biosensor fabricated on an ultrathin film. Photograph of the flexible device conformably attached onto the. (ii) Human wrist and (iii) artificial eyeball. (iv) Stretchable biosensor can be stretched with the activity of the human body. Adapted with permission from (Wang et al., 2020b). Copyright© 2020, MDPI. **(C)** (i) Schematics for the biochemical functionalization steps. (ii) Illustration of the PEI-modified GFET-based aptameric nanobiosensor. (iii) SEM image of the graphene conducting channel. Scale bar: 10 μm. (iv) The ultrathin flexible aptameric nanobiosensor is folded around a metal needle with a radius of 0.5 mm. Photograph of the stretchable aptameric nanobiosensor extended from 0% (v) to 120% (vi). Adapted with permission from (Hao et al., 2022). Copyright© 2022, ACS. **(D)** (I) Synthetic protocol of flexible graphene-based aptasensor on PEN film. (ii) Chemical reactions among 1,5-diaminonaphthalene (DAN), glutaraldehyde (GA) and the aptamer (3'-amine-TTC TTT CTT CCC CTT GTT TGT-C10 carboxylic acid-5'). (iii) Interaction of Hg^2+^ ions with thymine base pairs in the aptamer immobilized on the surface of the modified graphene layer. Adapted with permission from (An et al., 2013). Copyright© 2013, ACS.

In 2012, Oh Seok Kwon et al. firstly proposed that conducting polymers containing heteroatoms could be used to prepare doped graphene and successfully fabricated nitrogen-doped few-layer graphene (PPy-NDFLG) from polypyrrole (Figure 2A). They integrated the PPy-NDFLG with RNA aptamers specific to anti-vascular endothelial growth factor (VEGF) onto a flexible field-effect transistor (FET) platform based on a polyethylene naphthalate (PEN) substrate for electronic control. This work was the first to use nitrogen-doped graphene to manufacture a flexible FET-based aptamer sensor for the detection of VEGF as a cancer biomarker, and a detection limit of 100 fM was achieved (Kwon O S et al., 2012). In 2015, Cheng et al., 2015. Prepared a PDMS-supported GFET gated in phosphate-buffered saline (PBS) with an Ag/AgCl reference electrode solution. The highlight of this work was the use of a flexible PDMS substrate modified with APTES to form an amino-group-ended surface, graphene nanosheets were then self-assembled by covalent bonding with the terminal amino group on the PDMS substrate. The device was subsequently utilized for the label-free identification of the tumor marker alpha-fetoprotein (AFP), with a sensitivity threshold extending to 300 ng/mL (Ju C et al., 2015). In the same year, Sidra Farid et al. fabricated a F-GFET sensor also on PDMS substrate for the detection of interferon-gamma (IFN-γ), a biomarker for pneumonia and cancer. In this work, a DNA aptamer probe was employed to achieve precise and specific detection. The flexible sensor demonstrated remarkable sensitivity, enabling the detection of IFN-γ protein across a wide concentration range from nanomolar to micromolar levels, with an exceptional threshold as low as 83 pM (Farid S et al., 2015).

In 2017, Yang et al. developed a F-GFET sensor with a sensing element composed of a graphene nanomesh (GNM) featuring 3 nm pores. The GNM is a continuous two-dimensional graphene nanostructure with a high density of holes punched in the basal plane, introducing lateral confinement and improving the on/off ratio. Additionally, the graphene carrier concentration and mobility can be adjusted to enhance the sensor’s performance. By modifying the GFET with an aptamer, the sensor successfully detected human epidermal growth actor receptor 2 with a minimum detectable level of 0.6 pM (Yang Y et al., 2017). In 2018, Hao et al. engineered a GFET sensor incorporating oligonucleotides as the functional group on a 125 µm thick PEN substrate. By using VR11 DNA aptamers with high specificity to TNF-α, they investigated the effects of substrate bending on the equilibrium dissociation constant between the oligonucleotides and the biomarker as well as the graphene transconductance. This work demonstrated that the sensor could specifically respond to changes in TNF-α concentration within 5 min and an ultra-low detection capability o 26 pM in a repeatable manner (Hao Z et al., 2018). In 2019, Ziran Wang et al. fabricated a flexible and stretchable GFET sensor on a 2.5 μm Mylar substrate. Owing to its excellent flexibility, the sensor can conform to non-planar surfaces such as human skin or contact lenses and withstand large bending, twisting, and stretching deformations without significant mechanical damage, while maintaining consistent electrical performance. TNF-alpha was used as the target analyte, and a highly precise measurement down to 5 pM was achieved (Wang Z et al., 2019). The same group developed a F-GFET biosensor composed of graphene-Nafion composite. The graphene-Nafion composite film minimizes nonspecific adsorption and endows the biosensor with regenerability. This biosensor can detect cytokine storm biomarkers, including IFN-γ, in undiluted human sweat with a lower bound for detection at 740 fM Also, experimental results demonstrated that the biosensor maintained a consistent sensing response during regeneration and wrinkling tests without mechanical damage (Wang et al., 2020a). The same group also engineered a wearable and deformable F-GFET sensor on a ultrathin 2.5 μm thick substrate with a high mechanical durability. The authors used wet transfer and lithography process method to transfer graphene and gold electrode onto the substrate. The biosensor achieved a specific and sensitive detection of inflammatory cytokines TNF-a and IFN-γ, with detection limits of 2.75 and 2.89 pM, respectively (Figure 2B). This highly deformable biosensor may provide stable and sensitive detection of human cytokines, and is promising for the development of wearable biosensing systems (Wang et al., 2020b). Build on this work, Hao et al. developed a dual-channel F-GFET biosensor that enables multiplex detection of biomarkers. In this work, IFN-γ, TNF-a, and IL-6 in biological fluids were characterized in parallel under 7 min. The authors also integrated a customized Android application to potentially allow on-site detection (Hao Z et al., 2021).

In 2022, another aptamer-based F-GFET sensor was engineered by Hao and colleagues to rapidly detect hemoglobin in undiluted biological fluids. The sensor uses polyethyleneimine (PEI) as a low-cost linking molecule for the immobilization of aptamers. The experimental results indicate that the graphene sensor modified with PEI can respond to changes in hemoglobin concentration within 6–8 min, with a minimal measurable quantity of 10.6 fM in 1× PBS, 14.2 fM in undiluted serum, and 11.9 fM in undiluted urine, respectively. Additionally, the optimal PEI modification concentration was determined to be 0.4 μM based on comparison experiments of hemoglobin detection in undiluted serum. (Figure 2C). Therefore, this sensor has potential to accurately monitor hemoglobin in a clinical setting (Hao Z et al., 2022). In the same year, Laliberte et al. fabricated an F-GFET biosensor on a 25 μm thick polyimide substrate. By depositing a 50 nm thick silicon dioxide layer, the graphene was transferred to a relatively flat surface to ensure its high mobility. The authors showed that the silicon dioxide layer did not affect the biosensor’s flexibility, and coating the Kapton film with SiO_2_ significantly improved the transconductance and consistency of the device, and through continuous monitoring of IL-6 and real-time detection with a sensitivity threshold of 10 pM, this biosensor exhibited promising potential as a highly functional wearable device. (Laliberte K E et al., 2022).

### 3.2 F-GFET for other biomarkers detection

Compared to proteins, the application of F-GFET biosensors in detecting other types of biomolecules is far less common. In 2012, Yeon Hwa Kwak et al. developed a flexible glucose GFET sensor on a PET substrate, which exhibited ambipolar transfer characteristics. In its planar state, the sensor was capable of detecting glucose molecules in the range of 3.3–10.9 mM in PBS, with a detection limit of 3.3 mM. Even under deformation, the sensor was able to fit the model curve well and provide high-resolution, continuous real-time monitoring. This technology has significant potential for use in portable, wearable, and implantable glucose level monitoring applications (Kwak Y H et al., 2012).

In 2013, Ji Hyun An et al. reported the fabrication of a F-GFET sensor with rapid response to the heavy metal Mercury ion (Hg^2+^), which was designed for the detection and monitoring of the potential harm caused by Mercury ions to human health and the environment. (Figure 2D). The sensor used a 150 µm thick PEN substrate and DNA nucleic acid as the probe, achieving an ultra-low detection capability of 10 pM for Hg^2+^. The response time was less than 1 s and the sensitivity was 2-3 orders of magnitude higher than previous studies (An J H et al., 2013). In the same year, Oh Seok Kwon et al. engineered for the first time a large-scale patterned F-GFET immunosensor array on a PEN substrate. By densely stacking carboxylated polypyrrole nanoparticles (CPPyNP) on the graphene surface, a larger specific surface area was provided and HIV-2gp36 antigen (HIV-2 Ag) was immobilized on the particle surface for HIV detection, with a detection limit of 1 pM (Kwon O S, et al., 2013).

In 2022, Huang C et al. developed an ultra-flexible and transparent wearable GFET biosensor on a 1 μm thick PET substrate. The biosensor was designed to detect body fluid biomarkers, with a focus on L-cysteine and was able to detect L-cysteine in undiluted human sweat as well as artificial tears with a sensitivity threshold of 0.022 × 10^−6^ M, and 0.043 × 10^−6^ M, respectively. Considering its ultra-thin thickness and transparency, this F-GFET is expected to be used in applications such as contact lenses, which promotes the development of wearable biosensors in medical detection applications (Huang C et al., 2022). An overview of sensing performance of various GFET based biomolecule sensors are given in Table 1.

**TABLE 1:** An overview of sensing performance of various GFET based biomolecule sensors.

Ref	Sensor type	Target	Limit of detection	Sensitive range
Kwon et al., 2012	PPy-GFET	VEGF	100 fM	10fM-10nM
Cheng et al., 2015	PDMS-GFET	AFP	300 ng/mL	-
Farid et al., 2015	PDMS-GFET	IFN-γ	83 pM	2-100 µM
Hao et al., 2018	PEN-GFET	TNF-a	26 pM	50 pM-500 nM
Wang et al., 2019	Mylar-GFET	TNF-a	5 pM	50 pM-100 nM
Wang et al., 2020b	Mylar-GFET	TNF-a、INF-γ	2.75 pM、2.89 pM	0.2–500 nM 0.2–500 nM
Hao et al., 2022	PET-GFET	hemoglobin	10.6 fM	0.001nM–1000 nM
Laliberte et al., 2022	Kapton-GFET	IL-6	10 pM	10 pM-100 nM
Yang et al., 2017	PET-GFET	HER2	0.6 pM	0.0001–200 ng/mL
Kwak et al., 2012	PET-GFET	Glucose	3.3 mM	3.3–10.9 mM
An et al., 2013	PEN-GFET	Hg^2+^	10 pM	10 pM-100 nM
Kwon et al., 2013	PEN-GFET	HIV	1 pM	1 pM-10 nM
Huang et al., 2022	PET-GFET	L-cysteine	22 nM	0–4.8 nM

## 4 Conclusion and future research prospects

F-GFET biosensors have gained significant attention due to their potential for highly sensitive detection of biomolecule. GFET biosensors facilitate label-free, rapid and accurate detection of biomarkers, and flexible liquid-gated GFET biosensors exhibit excellent reusability, mechanical flexibility, and durability. Consequently, biosensors utilizing F-GFET technology hold immense potential in transforming into wearable devices for continuous health monitoring. However, the flexible liquid-gated GFETs are limited by the external gate electrode and the exposed properties of graphene that are susceptible to environmental contaminations, leading to limitations in application scenarios and inaccurate experimental results. Nevertheless, by regulating the charge movement in the graphene channel region through the back gate structure, it is possible to make its application scenario unrestricted, while effectively protecting the graphene from contamination through the dielectric layer, thus enhancing the sensitivity of flexible back-gated GFET biosensors. In the future, various back-gated GFET biosensors based on ultra-thin flexible substrates will become a promising real-time application device. At the same time, paper has the potential to become a flexible substrate for GFET biosensors, which can further reduce the manufacturing cost (Jia Y et al., 2020). In addition, With the development of virus real-time prediction analysis technology (Kukushkin V, et al., 2022) combined with AI technology on chips (Sun H, et al., 2022), research on F-GFET biosensors and the use of AI technology to enhance virus detection ability may enable the identification of various viral strains.
